# Editorial: Cardiovascular Adjustments and Adaptations to Exercise: From the Athlete to the Patient

**DOI:** 10.3389/fphys.2020.00187

**Published:** 2020-02-27

**Authors:** Antonio Crisafulli, Massimo F. Piepoli, Dick Thijssen, Pier Paolo Bassareo

**Affiliations:** ^1^The Sports Physiology Laboratory, Department of Medical Science and Public Health, University of Cagliari, Cagliari, Italy; ^2^Department of Cardiology, Polichirurgico Hospital G Da Saliceto, Piacenza, Italy; ^3^Radboud University Nijmegen Medical Centre, Nijmegen, Netherlands; ^4^University College of Dublin, Mater Misericordiae University Hospital, Dublin, Ireland

**Keywords:** stroke volume, heart rate, blood pressure, training, cardiac output

It is well-recognized that regular exercise confers protection against cardiovascular diseases, while, conversely, sedentary lifestyle is a risk factor. Trained individuals are usually less susceptible to cardiovascular diseases and adverse events than untrained ones. Moreover, from a morphological and physiological point of view, the cardiovascular apparatus is very different when comparing athletes and sedentary individuals.

Mechanisms through which physical activity provides with cardiovascular protection however are not well-understood. A well-known phenomenon is that some cardiovascular risk factors—such as high blood pressure, blood glucose dysregulation, obesity, muscle wasting, and high cholesterol—are positively affected by an active lifestyle. Moreover, regular exercise modifies genes expression and cardiovascular regulation and improves endothelial and platelet functions.

In this Research Topic we propose a few papers dealing with some beneficial effects of physical activity on the cardiovascular morphology, function, and regulation.

It has been demonstrated that the endothelial function is a valuable marker of cardiovascular health since it refers to the ability of the body to maintain the homeostasis of vascular tone. In their contribute to this Research Topic, Bisconti et al. were able to demonstrate that 8 weeks of knee extension exercise training improved endothelial cells response only in the femoral artery of the lower limb directly involved in the exercise, without affecting the endothelial response of the brachial artery, which on the contrary was not involved in the training protocol. Moreover, Boff et al. found in patients with type 1 diabetes that 8-week high intensity interval training leads to improvement in endothelial function and physical fitness. The described effect was greater than that afforded by moderate-intensity continuous training at a similar glycemic control (Boff et al.).

A precious review by Dombrowski et al. provides a *state of the art* about the cardiovascular regulation in patients with hypertension during exercise. Specifically, they described reflexes involved in cardiovascular dysfunction during effort in these subjects and concluded that much work is needed to fully understand what happens in hypertension in terms of cardiovascular regulation. Liang et al., using an animal model of hypertension (rats), provided evidence that neural mechanisms controlling blood circulation interact during exercise. In detail, there is an interactive relationship between central command and the exercise pressor reflex, which is inhibitory in nature. However, the neural occlusion between these central and peripheral pressor mechanisms is attenuated in hypertension.

Two studies, i.e., that by Gronwald et al. and that by Cardinale et al. dealt with the physiological effects of oxygen manipulation in inspired air. In the first investigation, by using heart rate variability, Gronwald et al. found that a hypoxic condition provoked a sympathetic activation compared to normoxia. In the second investigation, the Cardinale et al. reported that 6 weeks of hyperoxic-supplemented high intensity interval training led to only marginal gain vs. baseline in cycle performance in already trained cyclists, with no difference with respect to conventional training at sea level.

Sex-related differences in left ventricular morphology were addressed by Oláh et al. In a rat model of cardiac hypertrophy, the authors discovered that there is a more pronounced exercise-induced left ventricular hypertrophy in females as compared to males. However, only minor differences in left ventricular function were observed. Oláh et al. explained their results with molecular differences between genders.

Bernardi et al. assessed stroke volume during exercise from oxygen pulse in paralympic athletes. They reported significant differences in stroke volume as a consequence of the different health conditions. Moreover, they suggested that cardiac adaptations are possible also in paralympic athletes with spinal cord injury. Furthermore, they claimed that stroke volume can be predicted from O_2_ pulse measurements in these patients.

A research by Garnacho-Castaño et al. studied the slow component of VO_2_ and the exercise efficiency during resistance and endurance exercise. The authors reported a decrease in jump performance only after resistance training. Moreover, they claimed that gross efficiency could benefit from the eccentric phase of the resistance exercise.

Gajda et al. evaluated by echocardiography the heart function of swimmers after an ultramarathon relay. They observed that prolonged intense swimming did not affect left and right ventricular function, as no changes indicative of myocardial deterioration were detected 48 h after the event.

Finally, Currie et al. evaluated left ventricular structure with echocardiography in elite swimmers and runners. Their findings suggest enhanced early diastolic function in elite runners relative to swimmers. Authors attributed this result to faster left ventricular untwisting.

Taken together, all these studies strengthen the concept that exercise affects the cardiovascular system at various levels and in a complex way. Vascular reactivity, organs perfusion, nervous reflexes, cardiovascular regulation, genes expression, and molecules production by cells are all involved in the circulatory adjustments and adaptations to exercise. The regulation and the integration of many cardiovascular functions are significantly modified by exercise training.

## Author Contributions

AC, MP, DT, and PB conceived, wrote, and approved the editorial.

### Conflict of Interest

The authors declare that the research was conducted in the absence of any commercial or financial relationships that could be construed as a potential conflict of interest.

